# An Annular CMUT Array and Acquisition Strategy for Continuous Monitoring

**DOI:** 10.3390/s25216637

**Published:** 2025-10-29

**Authors:** María José Almario Escorcia, Amir Gholampour, Rob van Schaijk, Willem-Jan de Wijs, Andre Immink, Vincent Henneken, Richard Lopata, Hans-Martin Schwab

**Affiliations:** 1 Photoacoustics & Ultrasound Laboratory Eindhoven (PULS/e), Department of Biomedical Engineering, Eindhoven University of Technology, P.O. Box 513, 5600 MB Eindhoven, The Netherlands; a.gholampour@tue.nl (A.G.); r.lopata@tue.nl (R.L.); h.schwab@tue.nl (H.-M.S.); 2 XIVER MEMS Foundry, 5656 AE Eindhoven, The Netherlands; rob.van.schaijk@xiver.com; 3 Philips Innovation Services, 5656 AE Eindhoven, The Netherlands; willem.jan.de.wijs@philips.com; 4 Philips Engineering Solutions, 5656 AE Eindhoven, The Netherlands; andre.immink@philips.com (A.I.); vincent.henneken@philips.com (V.H.)

**Keywords:** capacitive micromachined ultrasonic transducer, CMUT, diverging waves, transmission beamforming, maternal monitoring, ultrasound imaging, transducer

## Abstract

In many monitoring scenarios, repeated and operator-independent assessments are needed. Wearable ultrasound technology has the potential to continuously provide the vital information traditionally obtained from conventional ultrasound scanners, such as in fetal monitoring for high-risk pregnancies. This work is an engineering study motivated by that setting. A 144-element annular capacitive micromachined ultrasonic transducer (CMUT) is hereby proposed for 3-D ultrasound imaging. The array is characterized by its compact size and cost-effectiveness, with a geometry and low-voltage operation that make it a candidate for future wearable integration. To enhance the imaging performance, we propose the utilization of a Fermat’s spiral virtual source (VS) pattern for diverging wave transmission and conduct a performance comparison with other VS patterns and standard techniques, such as focused and plane waves. To facilitate this analysis, a simplified and versatile simulation framework, enhanced by GPU acceleration, has been developed. The validation of the simulation framework aligned closely with expected values (0.002 ≤ MAE ≤ 0.089). VSs following a Fermat’s spiral led to a balanced outcome across metrics, outperforming focused wave transmissions for this specific aperture. The proposed transducer presents imaging limitations that could be improved in future developments, but it establishes a foundational framework for the design and fabrication of cost-effective, compact 2-D transducers suitable for 3-D ultrasound imaging, with potential for future integration into wearable devices.

## 1. Introduction

Monitoring of internal organs often requires repeatable, operator-independent measurements over time, as is the case in abdominal, cardiac, and obstetric care, to name a few. In the case of pregnancy, the pregnant body must undergo various adaptations to ensure safe gestation and delivery for both the fetus and the expectant individual [1]. Changes in vital signs often precede complications [2], therefore, monitoring of vital signs is crucial for effective obstetric care. Regular monitoring enables the early detection of complications, as in other internal organ monitoring cases, allowing a timely intervention to reduce the risk of morbidity and mortality in fetal and pregnant individuals [3,4,5,6,7,8].

For decades, cardiotocography (CTG) has been the standard of care for fetal monitoring before and during labor [9]. This technology facilitates the identification of suboptimal fetal heart rate behavior [10] by continuously recording ultrasound signals with a transducer placed on the abdomen of the pregnant individual. Uterine contraction signals can also be recorded using another transducer. Despite its advantages, CTG has shown limited reliability, is bulky, and requires frequent repositioning [11,12], which complicates repeated operator-independent use. These practical limitations motivate interest in solutions that allow stable placement and repeated measurements.

Ultrasound imaging provides valuable structural and motion information, but the form factors of devices currently integrated into the clinical workflow are generally unsuitable for continuous, hands-free monitoring. Therefore, the development of wearable ultrasound has become an important field of research for image-based monitoring of internal organs [13,14,15,16].

There are multiple challenges in the design and manufacturing of wearable ultrasound devices, such as costs, probe longevity, power consumption, and size [13]. The challenges become even more prominent in the case of 2-D transducers for 3-D ultrasound scanning, which has been of interest over the past decades in the obstetric field [17,18], as the large number of elements needed leads to increased complexity in manufacture and operation.

To address the challenge of high element count, researchers have proposed the strategic selection of a constrained subset of elements from a complete matrix array, thereby reducing the number of active channels [19,20,21]. In contrast, numerous design methodologies have been investigated for reducing channel counts in 2-D arrays. Row-column arrays, which comprise matrix elements that can only be addressed as entire columns or rows, have been proposed and repeatedly utilized in prior research [22,23,24,25]. Conversely, literature documents multiple low-element-density arrays, referred to as sparse 2-D transducers. For instance, Ramalli et al. [26,27], along with Vos et al. [28] and Martinez-Graullera et al. [29] illustrate the use of spiral arrays for three-dimensional scanning. Their studies encompass a broad range of topics, from optimization and simulation to practical implementation, while incorporating various beamforming methodologies. Furthermore, non-deterministic element positioning is also applicable to 2D aperture design, as proposed by Turnbull et al. [30], Diarra et al. [31], and Roux et al. [32], among others.

As an engineering step toward compact, hands-free ultrasound, we propose a 144-element annular capacitive micromachined ultrasonic transducer (CMUT) for 3-D ultrasound imaging. The device targets design constraints inspired by repeated obstetric assessments, but this work does not evaluate clinical outcomes. The proposed array is characterized by its compact size and cost-effectiveness. Although its geometry and low-voltage operation make it a candidate for future wearable integration, this study presents a benchtop prototype.

Unlike hand-held portable probes, such as Butterfly (Butterfly Network, Burlington, MA, USA), Lumify (Koninklijke Philips N.V., Eindhoven, The Netherlands), and Clarius (Clarius, Vancouver, BC, Canada), which stand out at operator-initiated point-of-care examination, the proposed transducer is designed for hands-free, operator-independent monitoring. This distinction is about intended use, not demonstrated clinical performance. The array design balances image uniformity within the field of view, moderate channel count, and practicality for future integration into a wearable device, rather than maximizing channel reduction alone, as in row-column arrays, or relying on nonuniform layouts, which can introduce angle dependence unless carefully optimized. However, it presents imaging challenges, including limited steerability, mainly attributed to the low number of elements and their positioning within the aperture.

For conventional arrays, numerous studies have shown that the quality of ultrasound images can be greatly enhanced by shaping the transmission beam through the manipulation of transmission delays [33,34,35]. Certain transmission beamforming methodologies can enhance image quality but often limit framerate. This is the case with focused transmissions that utilize multiple foci, where the beam is meticulously shaped to converge at a designated location, necessitating one transmission per voxel in extreme scenarios. Conversely, when priorities shift towards achieving high framerates, more straightforward transmission modes, such as plane or spherical waves, suffice, albeit at the expense of image quality. Our objective with the proposed transducer is to achieve good image quality without sacrificing framerate. This balance has been achieved through the utilization of various transmission schemes, with coherent compounding of diverging waves (DWs) prominently observed [36,37,38].

In the context of DW transmission, virtual sources (VSs) are meticulously placed above the surface of the transducer, and subsequent calculations are executed to determine the delays for each element. The employment of DW introduces a range of factors concerning the number and positioning of VSs, which affect the image quality and the attainable frame rate. In light of this, we propose the utilization of a Fermat spiral VS pattern for DW transmission and conduct a performance comparison with other VS patterns and standard techniques such as focused and plane waves. Our aim is to identify the optimal strategy that provides the most advantageous equilibrium between resolution, contrast, and frame rate for our 144-element annular CMUT, by benchmarking different VS patterns. Therefore, this paper addresses both the design and fabrication of the transducer while also presenting a quantitative assessment of various transmission strategies to enhance its imaging capabilities.

## 2. Materials and Methods

### 2.1. Transducer Design and Fabrication

The array follows an annular design with elements arranged around two concentric circumferences. The aperture is constructed in segments using multiple small dies that contain a subset of elements. We will refer to these segments as mutlets. Mutlets are a more robust and cost-effective way to manufacture transducers compared to the use of a single larger die because they mitigate yield risk by confining defects to small dies and enabling known-good-die assembly. Therefore, mutlet-based design represents an attractive technique for our envisioned low-cost solution.

**Figure 1:**
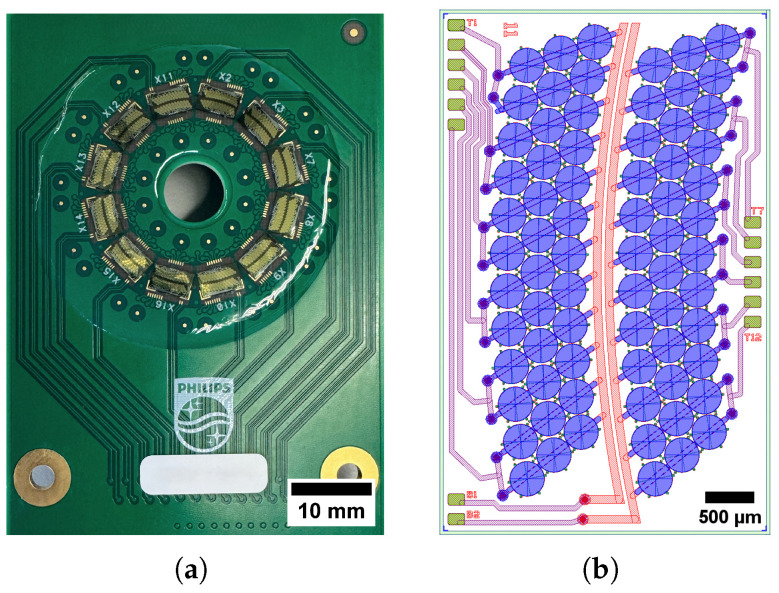
Proposed transducer. (**a**) Full aperture composed of 12 mutlets. (**b**) Schematic layout of a single mutlet illustrating the arrangement of six membranes per element across the 12 elements, with 6 elements distributed along each sub-arc of the circumference.

In total, 144 elements were distributed on 12 mutlets, with 12 arc-shaped elements each, as shown in Figure 1. The transducer design followed a two-stage procedure. First, we chose a suitable operational frequency for fetal monitoring and selected the element pitch and corresponding capacitive membrane size. Second, we optimized the full layer stack using finite element method simulations for mechanical and acoustical performance, considering collapse voltage, fractional bandwidth, transmit pressure, and frequency agility with bias variations. A schematic high-level process flow and performance of the CMUT technology can be found in [39]. For processing, surface micromachining was used, followed by dicing for mutlet singulation. The mutlets were then tested, and the selected ones were assembled onto a substrate using a pick-and-place technique.

Generally, CMUT elements consist of several capacitive membranes that are electrically connected. In this annular array, each element is composed of six membranes arranged in two columns, each with three membranes. Each membrane has a diameter of 350 μm and a center-to-center distance to the neighboring membrane of approximately 360 μm, so the element pitch is 720 μm.

The capacitive membranes are used in the collapse mode, which means that they function with a portion persistently in contact with the substrate [40]. This configuration achieves a maximum pressure of 1.4 MPa at a frequency of 3.2 MHz, exhibiting a sensitivity of 3.4 MPa/100V RF. Additional standard performance metrics are detailed in Table 1.

For this study, the array was assembled on a rigid PCB carrier to allow electrical access and benchtop acoustic characterization. This form factor is not a wearable device; rather, it provides performance data for a transducer intended for subsequent wearable packaging.

### 2.2. Transmission Sequence Design

To optimize the design of a transmission sequence capable of fully leveraging the potential of the transducer while preserving an appropriate frame rate, ultrasound simulations were conducted utilizing an in-house developed framework. In addition, the transmission schemes were implemented and evaluated through acquisitions.

#### 2.2.1. Simulation Method

There are several well-known and documented ultrasound simulation tools each with their own purpose, and therefore, their own advantages and limitations. In the case of Field II [41], volumetric imaging can be simulated, but circular elements must be constructed based on multiple small rectangles or triangles whose contributions are summed [42], which can represent a high computational load. In contrast, FOCUS, based on the method introduced by McGough in [43], can handle circular elements; however, it only simulates continuous wave pressures. This restriction limits its use to field analysis alone, and up to this point it does not include the capability to simulate pulse-echo signals [44]. Finally, the k-Wave toolbox [45] demonstrates the ability to model ultrasound transmission and reception with high physical accuracy. However, this capability simultaneously poses a limitation for the current study: the complex acoustic phenomena modeled by this tool entail a substantial computational load. This presents a significant challenge for conducting three-dimensional simulations on a volume of interest with dimensions equivalent to those of a pregnant abdomen. Furthermore, it proves inefficient for conducting numerous iterations of a design, which was requisite for this approach, especially given the degree of detail, which was redundant in this context.

In this study, the choice was to develop and use a custom simulation method to have increased flexibility and use a piston-based approach that closely resembles the capacitive membranes of a CMUT. The tool was developed in MATLAB (R2024b, The Mathworks, Natick, MA, USA) with a CUDA-based (Nvidia Corporation, Santa Clara, CA, USA) numerical time-of-flight simulation that models the propagation of ultrasound waves from circular pistons. At user-defined point scatterer locations, single scattering is assumed for simplicity and to reduce computational load.

The CUDA kernel loops over these point scatterers in the medium and accumulates their contributions in time for each receive channel. It also handles multiple transmit elements and models phenomena that affect the amplitude of the received signal. For a single scatterer, sc, let:

- $\left(z_{\mathrm{sc}},x_{\mathrm{sc}},y_{\mathrm{sc}}\right)$ be the position of a single scatterer.
- $\left(z_{\mathrm{TX},i},x_{\mathrm{TX},i},y_{\mathrm{TX},i}\right)$ be the position of the *i*-th transmitting element.
- $\left(z_{\mathrm{RX},j},x_{\mathrm{RX},j},y_{\mathrm{RX},j}\right)$ be the position of the *j*-th receiving element.

Then, the following distances can be computed:

(1) $$\begin{array}{cc} r_{\mathrm{TX}}^{\left(i\right)}\left(\mathrm{sc}\right) & =\sqrt{{\left(z_{\mathrm{sc}}-z_{\mathrm{TX},i}\right)}^{2}+{\left(x_{\mathrm{sc}}-x_{\mathrm{TX},i}\right)}^{2}+{\left(y_{\mathrm{sc}}-y_{\mathrm{TX},i}\right)}^{2}}, \end{array}$$

(2) $$\begin{array}{cc} r_{\mathrm{RX}}^{\left(j\right)}\left(\mathrm{sc}\right) & =\sqrt{{\left(z_{\mathrm{sc}}-z_{\mathrm{RX},j}\right)}^{2}+{\left(x_{\mathrm{sc}}-x_{\mathrm{RX},j}\right)}^{2}+{\left(y_{\mathrm{sc}}-y_{\mathrm{RX},j}\right)}^{2}}. \end{array}$$

with $r_{\mathrm{TX}}^{\left(i\right)}$ being the distance from the TX element *i* to the scatterer and $r_{\mathrm{RX}}^{\left(j\right)}$ the distance from the scatterer to the RX element *j*.

Having the precomputed transmit delay applied to element *i*, $\Delta t_{\mathrm{TX}}^{\left(i\right)}$, and the propagation distances $r_{\mathrm{TX}}^{\left(i\right)}$ and $r_{\mathrm{RX}}^{\left(j\right)}$ normalized by the speed of sound *c*, the time-of-flight $t_{\mathrm{TOF}}^{\left(i,j\right)}$ can be computed as

(3) $$t_{\mathrm{TOF}}^{\left(i,j\right)}=\Delta t_{\mathrm{TX}}^{\left(i\right)}+\frac{1}{c}\left[r_{\mathrm{TX}}^{\left(i\right)}\left(\mathrm{sc}\right)+r_{\mathrm{RX}}^{\left(j\right)}\left(\mathrm{sc}\right)\right].$$

The amplitude of the received signal is calculated by scaling the initial scatterer strength, $A_{1,\mathrm{sc}}$. The following factors can be taken into account for the amplitude scaling:

- *Spherical spreading* factor
  (4) $${\hat{P}}_{\mathrm{ss}}^{\left(i,j\right)}=\frac{1}{r_{\mathrm{TX}}^{\left(i\right)}\left(\mathrm{sc}\right)\;r_{\mathrm{RX}}^{\left(j\right)}\left(\mathrm{sc}\right)}.$$
- *Attenuation*, for which a uniform coefficient α may be specified, giving
  (5) $${\hat{P}}_{\alpha }^{\left(i,j\right)}=\exp\;\left[-\alpha \;t_{\mathrm{TOF}}^{\left(i,j\right)}\right].$$
- *Scatterer directivity*
  (6) $${\hat{P}}_{sd}=\frac{{\vec{r}}_{\mathrm{tx}}\;\cdot \;{\vec{r}}_{\mathrm{rx}}}{∥{\vec{r}}_{\mathrm{tx}}∥\;∥{\vec{r}}_{\mathrm{rx}}∥}.$$
  Scatterer directivity only applies to density scatterers, which behave as dipoles. For improved comprehension, only bulk-modulus scatterers, i.e., monopole-like scatterers, are modeled in the upcoming analysis.
- *Element directivity*, which is modeled with a Bessel-based scaling
  $${\hat{P}}_{ed}\left(\theta \right)=\frac{2\;J_{1}\;\left(\frac{W_{\mathrm{rel}}}{2}\sin\theta \right)}{\frac{W_{\mathrm{rel}}}{2}\sin\theta },$$
  where $J_{1}$ is the first-order Bessel function and $W_{\mathrm{rel}}$ is the element width relative to the center wavelength, so
  (7) $${\hat{P}}_{ed}\left(\theta \right)=\left\{\begin{array}{cc} 1, & \frac{W_{\mathrm{rel}}}{2}\sin\theta \;\approx 0,\\ \frac{2J_{1}\;\left(\frac{W_{\mathrm{rel}}}{2}\sin\theta \right)}{\frac{W_{\mathrm{rel}}}{2}\sin\theta },\; & \mathrm{otherwise}. \end{array}\right.$$

For the first-order Bessel function, a piecewise approximation based on its series expansion was implemented, as explained in Appendix A.

Putting together Equations (4) to (7), the received amplitude, $A_{\mathrm{sc}}^{\left(i,j\right)}$, can be determined:

(8) $$A_{\mathrm{sc}}^{\left(i,j\right)}=A_{1,\mathrm{sc}}\;{\hat{P}}_{\mathrm{ss}}^{\left(i,j\right)}\;{\hat{P}}_{\alpha }^{\left(i,j\right)}\;{\hat{P}}_{ed}\left(\theta \right)\;{\hat{P}}_{sd}.$$

The amplitude is subsequently assigned to the corresponding time index. To compute the weights for adjacent non-integer indices, a second-order polynomial is utilized. Consequently, a peak is created mimicking a sinc function centered at the specified non-integer time index. Let $t_{\mathrm{TOF}}^{\left(i,j\right)}$ represent the time-of-flight for TX element *i* to RX element *j*. Define

$$n_{\mathrm{TOF}}=\left\lfloor \frac{t_{\mathrm{TOF}}^{\left(i,j\right)}}{T_{s}}\right\rfloor ,\;T_{s}=\frac{1}{f_{s}}.$$

$$w_{1}=\frac{t_{\mathrm{TOF}}^{\left(i,j\right)}}{T_{s}}-n_{\mathrm{TOF}},\;w_{2}=1-w_{1},$$

$$w_{f,1}={\left(w_{1}^{2}-1\right)}^{2},\;w_{f,2}={\left(w_{2}^{2}-1\right)}^{2}.$$

In the sampled domain (n∈Z) this resembles:

(9) $$S_{A_{j}}\left[n\right]=\sum_{\mathrm{sc}}\;\sum_{i}A_{\mathrm{sc}}^{\left(i,j\right)}\left[w_{f,1}\;\delta \left(n-n_{\mathrm{sc}}\right)+w_{f,2}\;\delta \left(n-(n_{\mathrm{sc}}+1)\right)\right],$$

where $n_{\mathrm{sc}}$ is the integer arrival index for the scatterer sc. In continuous time, this is equivalent to:

$$S_{A_{j}}\left(t\right)=\sum_{\mathrm{sc}=1}^{N_{\mathrm{sc}}}\;\sum_{i=1}^{N_{\mathrm{tx}}}A_{\mathrm{sc}}^{\left(i,j\right)}\;\delta \;\left(t-t_{\mathrm{TOF}}^{\left(i,j\right)}\right).$$

The resulting signals are postprocessed in MATLAB by filtering the accumulated raw impulses with the transducer’s impulse response.

The simulation tool was benchmarked against results obtained by numerically solving the Rayleigh–Sommerfeld integral (RSI), and the $J_{1}\left(x\right)$ piecewise approximation was separately verified against an analytical solution to ensure accuracy and stability.

#### 2.2.2. Simulation Steps

For the optimization and evaluation of the proposed VS patterning and its comparison with other transmission schemes, the simulation tool described above was used. The speed of sound was established at 1540 m/s, and the simulation’s center frequency was configured at 2.7 MHz. The definition of three additional factors is fundamental to the simulation: aperture description, scatterer location, and transmission delay computation.

**Figure 2:**
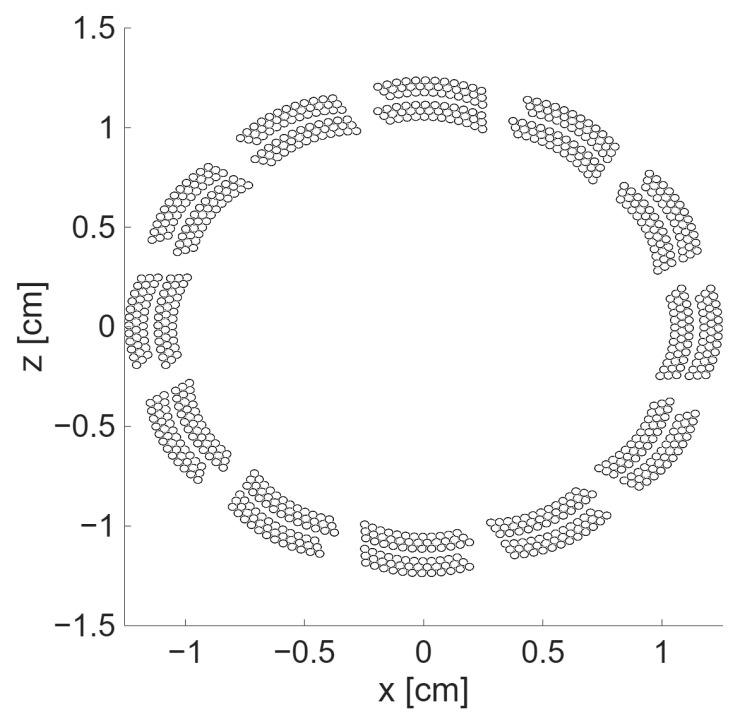
Transducer definition, membrane by membrane, as circular elements.

The *transducer aperture definition* was carried out meticulously with adherence to the physical configuration of the elements within the transducer, describing each capacitive membrane, as shown in Figure 2.

**Figure 3:**
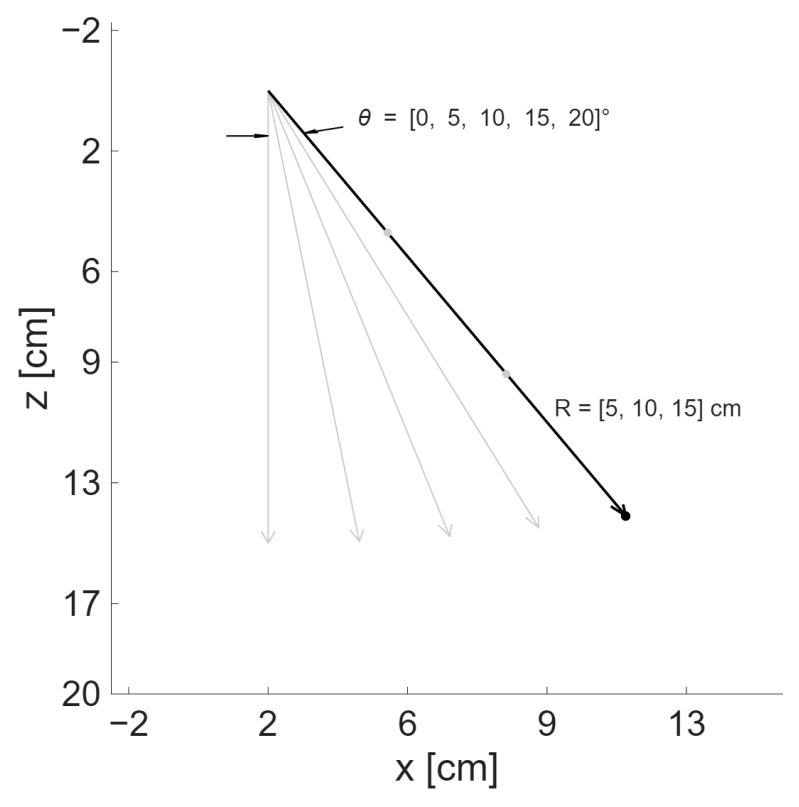
Different positions of scatterer for iterative evaluation of PSF. All combinations of θ and *R* were simulated and posteriorly evaluated.

For this study, a point spread function (PSF)-based analysis was chosen. Therefore, for each simulated transmission, a single scatterer was localized within the volume of interest. Since the envisioned application of the proposed aperture is fetal monitoring, a depth of imaging of at least 20 cm was selected, taking into account the mean fundal distance during the second trimester of pregnancy. In addition, at maximum depth, a minimum imaging angle of 20° was chosen to cover the lateral area where the fetal heart could be located. Therefore, for each transmission evaluated, the scatterer was iteratively moved in depth and lateral distance from the origin, as illustrated in Figure 3. The transducer is axisymmetric; therefore, the scatterer was moved only in the positive x-axis.

For each simulated acquisition, transmission delays were computed. These delays depended on the intended transmission sequence and the target focus point, in the case of focused transmission. In this study, we propose the placement of VSs for DW transmission following a Fermat’s spiral pattern to exploit its reduced periodicity, which has been observed to lead to lower sidelobe levels and grating-lobe suppression in various fields, such as antenna array and physical ultrasound source design [29,46].

To elucidate the benefits associated with this mode of transmission, simulations of focused and steered transmissions were conducted for each scatter position. This approach typically yields superior imaging performance, albeit at the expense of a reduced frame rate due to the requirement for multiple focal points in a volume of interest pertinent to the given application of this study. Furthermore, a single plane-wave transmission was simulated, which generally results in inferior image quality yet offers an enhanced frame rate. Finally, more traditional arrangements of VSs for DW transmission were also simulated for reference. In this instance, adhering to the rationale of gridded VSs typically employed in matrix arrays, the VSs were organized in one circle and two concentric circles, thereby replicating the element distribution of the proposed aperture.

For each of the transmission sequences mentioned above, *transmission delays were computed* as follows:

- For the three VSs patterns, the transmission delays corresponding to each VS were computed and assigned as independent transmission events.
- For the focused transmission, the delays to focus and steer the beam to the location of the point scatterer were computed for each element. To reduce computational load, only one focus point was defined, which was collocated with the scatterer.
- For the plane wave, as for the focused transmission sequence, only one angle was used, corresponding to the angle used to position the scatterer.

Given a pattern, the number of VSs, $n_{vs}$, their maximum distance from the transducer, $d_{v_{max}}$, and the maximum angle of divergence, $\alpha_{v_{max}}$, must be determined. Calculating $\alpha_{v_{max}}$ ensures that the edge elements are physically capable of transmitting at the given angle; therefore, this is determined by the directivity of the element. Details of the estimation of $\alpha_{v_{max}}$ are given in Appendix B.

However, when the VSs are placed too far behind the transducer, the wavefront can be too flat, resembling more a plane wave rather than a DW. To avoid this, $d_{v_{max}}$ can be calculated as the distance at which a curved wavefront becomes effectively planar:

$$d_{v_{max}}=\frac{A^{2}}{\lambda },$$

where *A* is the aperture size and λ is the wavelength. In our case, $A^{2}\gg \lambda$, so $d_{v_{max}}$ is very large. In this study, the effect of the distance between the transducer and the VSs was not studied and was arbitrarily set to 20 mm.

**Figure 4:**
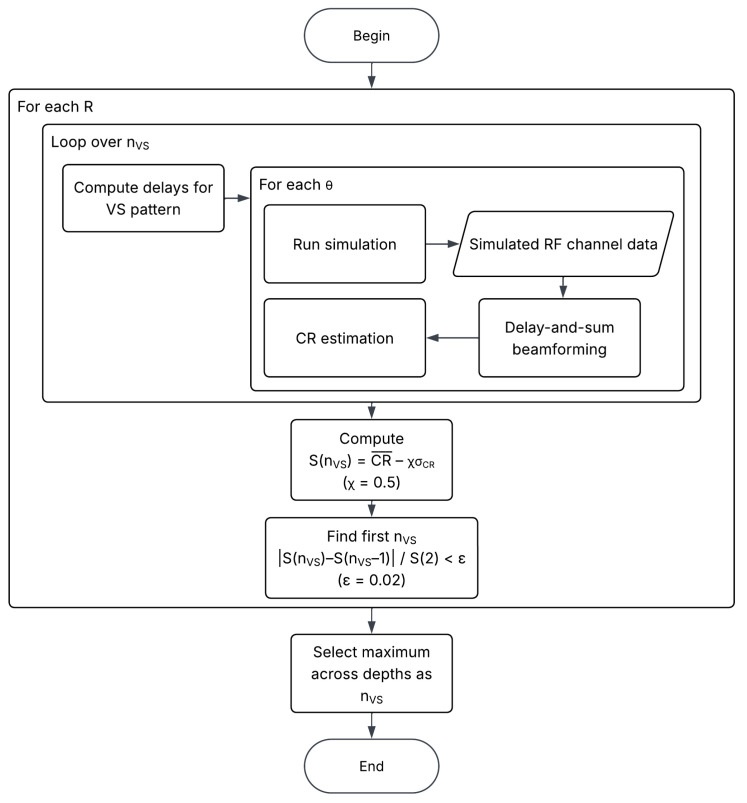
Flowchart depicting steps that were followed for the selection of the optimal $n_{vs}$ for each VS pattern included in the study.

Ultimately, the estimation of the optimal $n_{vs}$ was performed through an iterative increase and evaluation process of the contrast ratio (CR). This process yielded several values that were synthesized for enhanced interpretability using

$$S\left(n_{vs}\right)=\bar{\mathrm{CR}}-\chi \sigma_{\mathrm{CR}},$$

with χ representing a penalization factor, which was set to 0.5, and $\sigma_{\mathrm{CR}}$ the standard deviation of the CR values. The computations for both $\bar{\mathrm{CR}}$ and $\sigma_{\mathrm{CR}}$ were carried out according to $n_{vs}$. The methodology employed for the selection of $n_{vs}$ is systematically outlined in a flowchart presented in Figure 4.

#### 2.2.3. Evaluation Metrics

In order to evaluate each transmission scheme, four metrics were computed for each position of the scatterer along the PSF x-axis profile in the particular z-plane in which the scatterer was located:

1. Full width at half-maximum (FWHM): Lateral distance (x-axis) when the profile amplitude drops to −6 dB. FWHM assesses the resolution of the system, so the smaller the value, the better the performance.
2. CR: Ratio of the average envelope amplitude in a volume of interest to the average envelope amplitude of the background. The volume of interest was defined as a cube of dimensions given by the FWHM, centered at the known scatterer position. The remaining reconstruction volume was set as background. CR is used to estimate the visibility of the targeted structure and is expected to be maximized for improved detectability.
3. Peak sidelobe level (PSL): Amplitude of the largest sidelobe relative to the main lobe computed on the PSF x-axis profile. PSL offers insights into the apex intensity emanating from sidelobes, which is undesirable and therefore aimed to be minimized.
4. Integrated sidelobe level (ISL): Ratio of integration over the sidelobe region with respect to the integration of the main lobe section of the PSF x-axis profile. High ISL reduces contrast, as it represents the total energy in the sidelobes with respect to the energy of the main beam.

To evaluate the significance of the differences between the results of the different sequences, different statistical tests were used. Initially, the assumptions of normality and homoscedasticity were examined via the Shapiro–Wilk test and the Levene test, respectively. In instances where all transmission results at a specified *R* were identified as normal and homoscedastic, repeated measures ANOVA was used for global assessment, succeeded by the *t*-test with Holm–Bonferroni correction for paired comparisons. In contrast, if non-normality was observed in at least one of the transmissions within the specified *R*, the Friedman test was applied for group analysis, and the Tukey–Kramer test with Holm–Bonferroni correction was used for paired testing. In scenarios where sequence results were normal yet nonhomoscedastic, nonparametric tests were conducted due to the reduced sample size.

### 2.3. Experimental Validation

The experimental acquisitions were performed using the proposed transducer connected to a Verasonics Vantage 256 system. The implementation of the optimized VS distribution adhering to a Fermat’s spiral was employed to establish the transmission delays. The scanning process was carried out employing the Multi-Purpose Multi-Tissue Ultrasound Phantom (040GSE, CIRS, Norfolk, VA, USA), composed of Zerdine, a solid elastic tissue-mimicking hydrogel incorporating multiple wire and cyst inclusions.

The acquisitions were analyzed by calculating the FWHM and CR, as indicated in Section 2.2.3, for each of the wires. For the CR, a certain region was selected as a background and used for all evaluations. The CR metric in this case is used to assess the possible visibility deterioration of the threads due to depth-dependent attenuation. For evaluation purposes, the locations of the z and x coordinates of the wires were manually determined. Seventy-five percent of the y-planes were used to create the volumetric mask for the wires. In addition, the upper and lower limits of a slab located at the base of the acquired volume were selected to serve as the background for the CR evaluation. This background selection does not allow for the CR to serve as a metric for wire visibility, but rather allows for a fair relative comparison of wire intensities over depth.

## 3. Results

### 3.1. Simulation Method Validation

**Figure 5:**
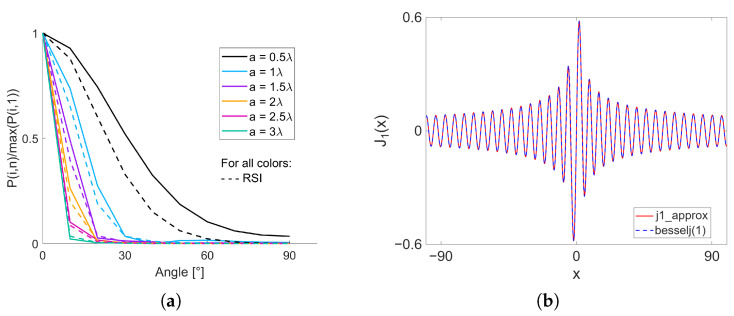
Proposed simulation framework validation results. (**a**) Plot summarizing comparison of the peak amplitude of the simulated RF channel data using the proposed simulation tool and an implementation of the RSI. Piston width, denoted by *a*, was varied from 0.5λ to 3λ. (**b**) Comparison of results from piecewise $J_{1}\left(x\right)$ approximation (j1_approx, in red) and from the analytical solution from MATLAB (besselj(1), in blue) from an argument range −100≤x≤100.

The simulation pulse peak amplitudes closely matched the RSI values across angles and piston widths (*a*), with 0.089 as the highest mean absolute error (MAE). Figure 5a synthesizes the results obtained for *a* that vary from 0.5λ to 3λ, while the MAE for each *a* are reported in Table 2. As can be observed in the plot, the simulation values show a high resemblance to the corresponding RSI results, especially when moving towards larger *a*, which is confirmed by the MAE values.

Furthermore, the outcomes of the $J_{1}\left(x\right)$ piecewise approximation, used to model the scaling of amplitude as a consequence of the directivity of elements, along with the results of the analytical solution of $J_{1}\left(x\right)$ are plotted in Figure 5b. The $J_{1}$ approximation aligned closely with the analytical solution, as proved by a root mean square error of 0.005 [-] in the wide range of *a* that was evaluated.

### 3.2. Transmission Sequence Design

#### 3.2.1. Selection of $n_{vs}$ for Each VS Pattern

**Figure 6:**
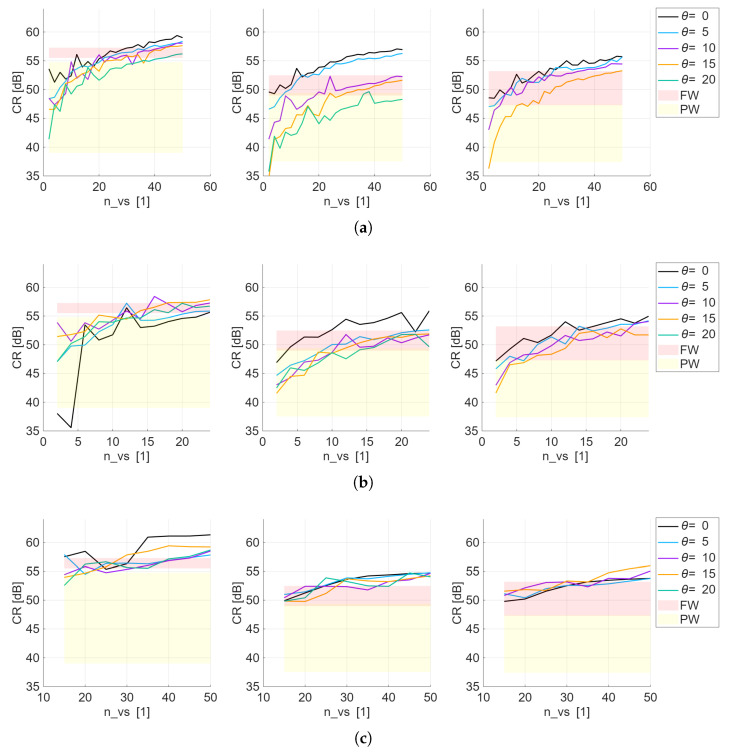
CR curves obtained with the VSs following (**a**) a circular arrangement, (**b**) two concentric circular arrangement, and (**c**) a Fermat’s spiral arrangement, as the number of fired VSs, $n_{vs}$, increases. Each column corresponds to a point-scatterer *R* of 5 cm, 10 cm, and 15 cm, respectively. The five colored traces are the five θ studied. The red and yellow shaded bands reproduce the reference CR obtained with a fully focused transmit (upper band) and a plane wave transmit (lower band), respectively. A curve that rises into the red band meets or exceeds the focused baseline; one that falls into the yellow band performs only as well as the plane wave case.

The resulting CR scores when varying $n_{vs}$ for all VS patterns are shown in Figure 6. The red and yellow panels, representing the range of results for all angles when employing focused and plane wave transmission, respectively, are displayed to allow understanding of the performance of the different DW sequences with respect to these two baseline transmissions. It can be observed that interpreting the raw CR results could be challenging due to the resulting curves’ behavior, especially in the case of the circular and two concentric circular arrangements of virtual sources. This highlights the importance of an additional processing step before selecting $n_{vs}$ for each pattern, which in this case is represented by the calculation of $S(n_{vs})$.

**Figure 7:**
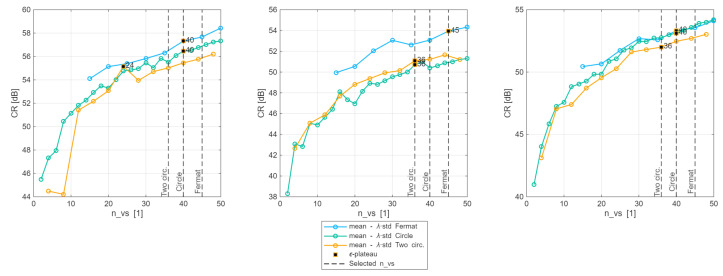
Choice of optimum $n_{vs}$ per arrangement type using the $S(n_{vs})$ score over the five θ. The blue line corresponds to the circle arrangement, orange corresponds to the two circles arrangement, and green to the Fermat’s spiral. Each panel corresponds to a point-scatterer *R* of 5 cm, 10 cm, and 15 cm, respectively. The black and orange square shows the first ε-plateau point, i.e., the smallest $n_{vs}$ that attains at least 98% (ε=2%) of the global maximum CR. The dashed line shows the chosen $n_{vs}$: the maximum ε-plateau from all *R*.

In Figure 7, the resulting $S(n_{vs})$ curves for all VS patterns are depicted. Please note that the first and last $n_{vs}$ that were evaluated vary according to the pattern. The maximum $n_{vs}$ was set to 50, to obtain a reasonable frame rate. However, for two concentric circles, a minimum of four VSs was needed to have at least two on each circumference. VSs were increased in steps of two per circumference; therefore, a maximum $S(n_{vs})$ of 48 was reached. In the case of the Fermat spiral, around 15 VSs were needed to start observing a pattern, so this was set as the starting point.

Regarding the ε-plateau, it can be noted that the configuration of two concentric circles necessitates the least amount of $n_{vs}$ to achieve this point, followed by the singular circumference and ultimately Fermat’s spiral. It is logical to anticipate that a larger *R* will require a larger number of VSs to reach the ε-plateau. Curiously, for Fermat’s spiral, the middle *R* demanded more $n_{vs}$ to attain the plateau compared to the maximum *R*, and the circumference required more $n_{vs}$ for the initial evaluated *R* than for the middle one.

**Figure 8:**
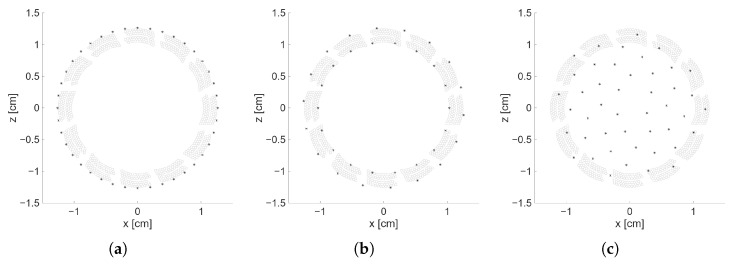
Distribution of VSs of each pattern after selection of optimal $n_{vs}$. (**a**) VSs following a circular arrangement ($n_{vs}$ = 40). (**b**) VSs following two concentric circles arrangement ($n_{vs}$ = 36). (**c**) VSs following a Fermat’s spiral arrangement ($n_{vs}$ = 45). For all panels, the gray circles represent the element’s capacitive membranes and the black filled hexagram corresponds to the VSs.

**Figure 9:**
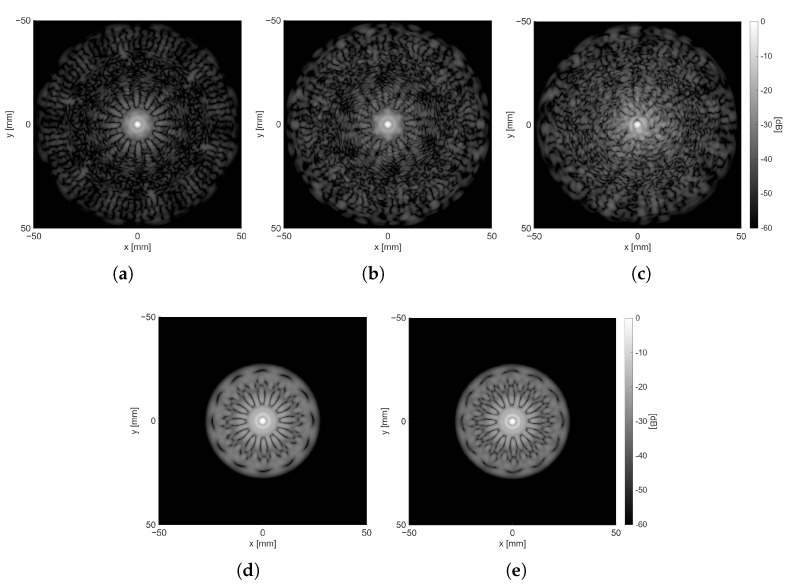
The xy-plane at scatter location in z-axis when R=10 cm (for θ=0) with a dynamic range of 60 dB. (**a**) VSs following a circular arrangement ($n_{vs}$ = 40). (**b**) VSs following two concentric circles arrangement ($n_{vs}$ = 36). (**c**) VSs following a Fermat’s spiral arrangement ($n_{vs}$ = 45). (**d**) Plane wave transmission. (**e**) Focused wave transmission.

**Figure 10:**
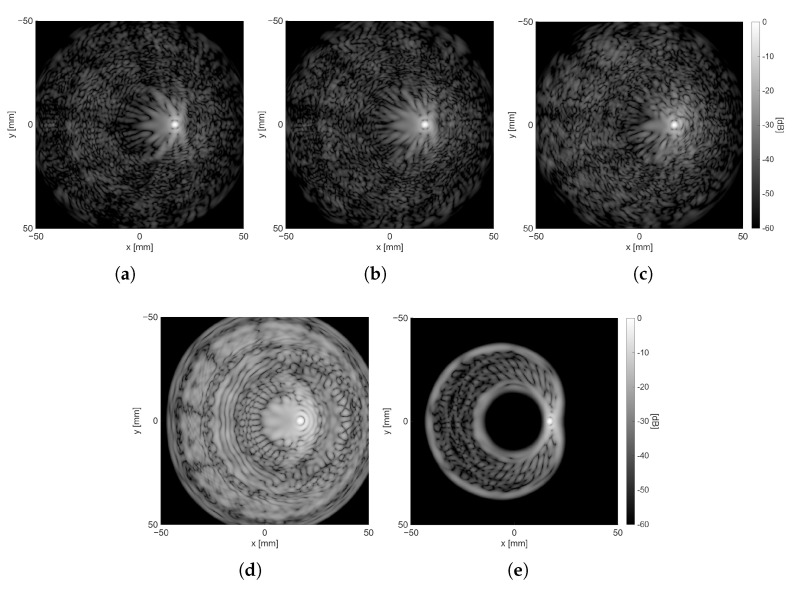
The xy-plane at scatter location in z-axis when R=10 cm for θ=10° with dynamic range of 60 dB. (**a**) VSs following a circular arrangement ($n_{vs}$ = 40). (**b**) VSs following two concentric circles arrangement ($n_{vs}$ = 36). (**c**) VSs following a Fermat’s spiral arrangement ($n_{vs}$ = 45). (**d**) Plane wave transmission. (**e**) Focused wave transmission.

Based on the results presented, the optimal $n_{vs}$ per pattern was selected and summarized in Table 3. Figure 8 shows the final distribution of VSs for each pattern, while Figure 9a–c presents an example of the reconstruction of the xy-plane for R=10 cm, and θ=0° at z=10 cm, hence the *z*-coordinate of the position of the scatterer. Reconstructions of the same plane for the focused and plane wave transmission cases are shown in Figure 9d–e for reference. Similarly, Figure 10a–e shows the same scenario but with θ=10° to show the differences when the scatterer is off-center.

#### 3.2.2. Performance Evaluation of Transmission Schemes

**Figure 11:**
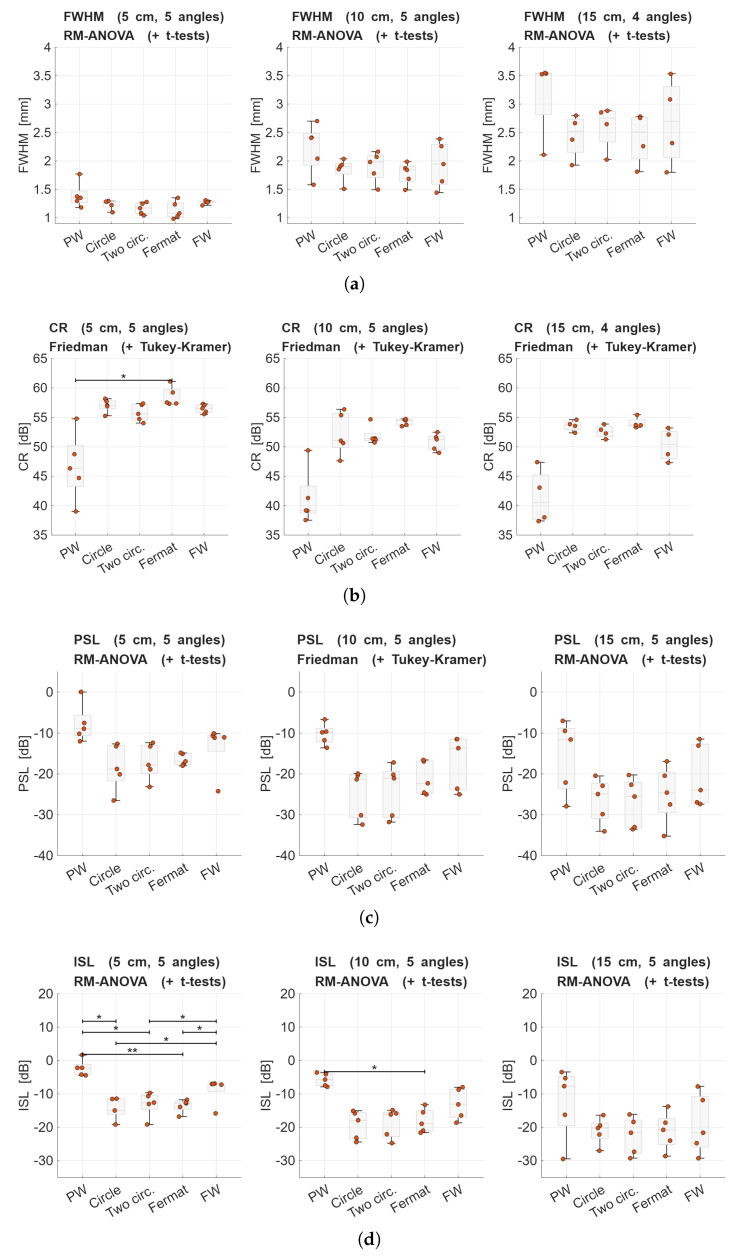
Box plot representation of the results of optimized VS patterns and baseline transmissions at different *R*. (**a**) FWHM, (**b**) CR, (**c**) PSL, and (**d**) ISL achieved by each transmit sequence. Orange dots are the individual metric results for the different steering angles evaluated. An asterisk denotes the pairwise significant difference (0.01≤p<0.05). For all panels, PW stands for plane wave whereas FW is short for focused wave.

The optimized VS patterns and two baseline transmissions comparisons based on the four performance metrics are summarized in Figure 11. It can be observed that the three DW sequences consistently exhibit reduced and less dispersed FWHM results. Similarly, this trend is noted for the CR, wherein these sequences demonstrate enhanced performance, particularly when VSs are configured in accordance with a Fermat’s spiral distribution, attaining the highest CR values across all depths, closely followed by the other VS patterns. Regarding the PSL and ISL, the two configurations based on circular arrangements display the best performance out of all studied sequences. This can be further understood by examining the PSF examples in Figure 9, which show that the DW sequences manifest lower sidelobe levels, although with unfavorable contributions spanning a wider area compared to, for example, the focused transmission.

All metrics for the different *R*s that were evaluated were found to have a global significant difference (see Table 4). For pairs with significant differences according to the post hoc tests, an asterisk (*) was added to pairs with significant differences 0.01≤p<0.05. In contrast, for pairs with p<0.01, two asterisks (**) were used. FWHM and PSL do not show a significant difference between any of the possible pairs. Across the different VS patterns, visual inspection of the results shows differences, but they are not statistically significant in most cases, excluding CR at R=5 cm between the Fermat’s spiral and the plane wave transmission and a plethora of pairwise differences for ISL.

#### 3.2.3. Evaluation of Experimental Data

**Figure 12:**
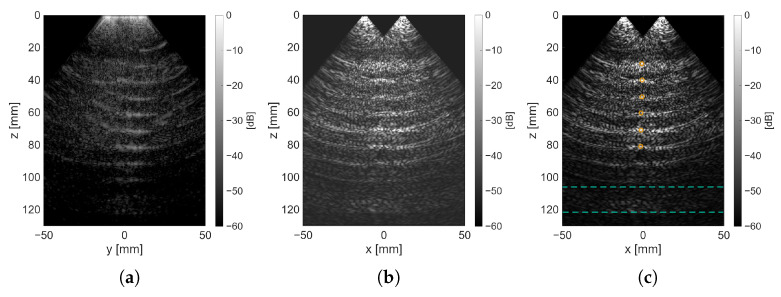
Acquisition plane reconstruction. (**a**) Post-processed zy-plane MIP of x-axis showing the length of wires at different depths. (**b**) Post-processed center slice in y-axis of zx-plane showing a cross-section of the wires. (**c**) Overlay of mask contours of wires (in orange) and slab for background (in blue).

Figure 12a shows the center zx-plane of the reconstructed volume data, while Figure 12b depicts the zy-plane of a maximum intensity projection (MIP) over the *x*-axis. The wires in the phantom were roughly aligned along the *y*-axis. For visualization purposes, the contrast was enhanced using a sigmoid S-curve. Note the six reflections at different depths from the distinguishable wires. It should be considered that, in contrast to the simulation setup, in this case the background is not fully anechoic. The sidelobe contributions, in addition to the background scatterers, give rise to the fanning reflections on the sides of each wire.

**Figure 13:**
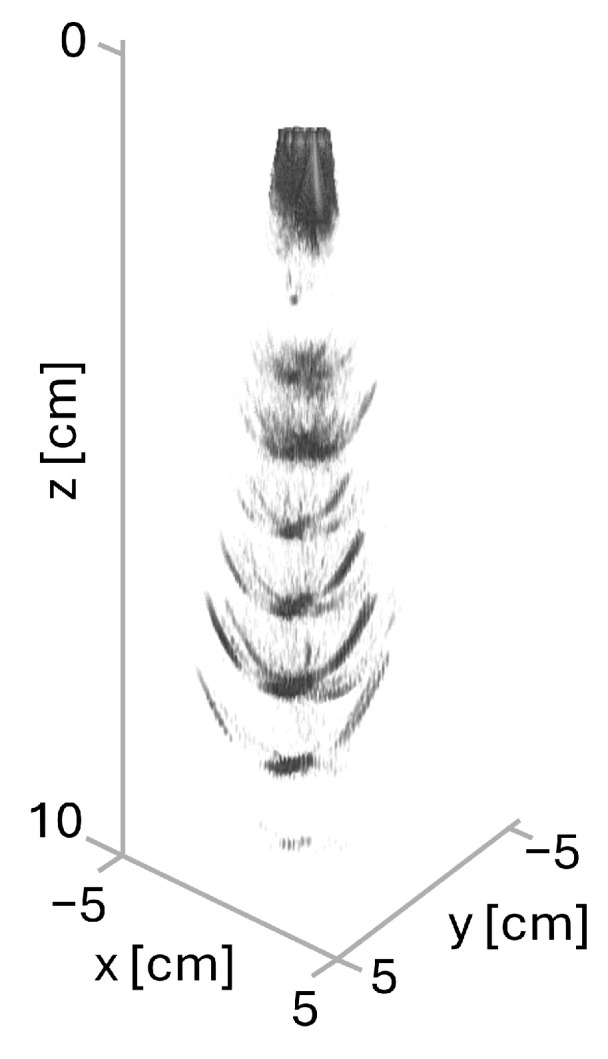
A 3-D ultrasound volume rendering of a CIRS phantom acquisition. Opacity is mapped to echo strength so weak speckle becomes transparent and only strong reflectors remain, with depth increasing downward. The effect of side lobes is evident, as the threads appear as curved planes rather than straight tubes.

The locations of the wire and the slab selected in the center plane are shown in orange and blue, respectively, in Figure 12c. In addition, a 3-D ultrasound volume rendering is shown in Figure 13. The intensity map was adjusted to show only strong reflections that come mainly from the wires.

The results of the metrics for each of the wires are summarized in Table 5. For the last identified wire, it was not possible to determine the FWHM since the first side lobe peak rose before the main lobe could drop −6 dB.

## 4. Discussion

This study aimed to engineer a compact and cost-effective 2-D ultrasound transducer, representing a step toward developing compact, autonomous ultrasound technology, suitable for future integration into wearable devices for image-based monitoring. The proposed CMUT array adopts a sparse annular mutlet-tiled geometry that balances aperture size with a moderate channel count, lowering interface complexity (fewer cables, interconnects, and no ASIC required) while preserving non-separable 3-D apodization and focusing. The annular symmetry yields an angle-uniform PSF across steering angles, and the mutlet modular architecture enables known-good-die assembly and selective replacement, which can improve manufacturing practicality and yield. Compared with other sparse 2-D arrangements (e.g., spiral or stochastic layouts), the concentric-ring topology offers regular routing and layout with a large effective aperture, providing a pragmatic trade-off between image uniformity and cost/complexity.

While the transducer’s compact design supports integration into wearable systems, achieving practical wearability requires biocompatible encapsulation and a skin adhesive for comfort and coupling, strain-relieved cabling or wireless links, continuous use thermal and electrical safety verification, and long-duration monitoring power supply, to name a few considerations. These packaging and system engineering steps are beyond the scope of the present transducer study but are planned for future work.

Moreover, its sparse configuration introduces imaging limitations, particularly in steerability. To address these challenges and enhance imaging performance, we investigated diverging wave transmissions using VSs, including a Fermat’s spiral arrangement, and compared them with conventional focused and plane wave strategies.

To optimize the number of VSs per arrangement and to facilitate subsequent comparisons between different transmission sequences, a simplified and versatile simulation framework, enhanced by GPU acceleration, has been developed. The results obtained from the RSI validation show a strong correspondence with the expected outcomes, particularly as *a* approaches at least 1λ. The dimensions of the elements directly influence the pitch of the aperture, which plays an important role in the presence of grating lobes and, therefore, is preferred to be equal or smaller than 0.5λ. The results of the validation indicate that simulations utilizing transducers with an ideal pitch may not be as effective as those designed with larger pitches. On the other hand, the analytical validation of the $J_{1}\left(x\right)$ piecewise approximation, employed to mimic the amplitude scaling due to the directivity of the element, demonstrated good performance and stability for a wide range of function arguments.

Figure 9 illustrates that the DW sequences extend the influence of sidelobes over a broader spectrum, while the impact of secondary lobes on baseline transmissions is confined to a smaller radius surrounding the point scatterer’s position. This phenomenon is particularly significant when the scatterer is not precisely positioned at the center of the transducer along the x and y axes, as depicted in Figure 10, where the disparities between the DW and baseline sequences in terms of sidelobe contributions are distinctly observable. A notable aspect that intensifies when transitioning from Fermat’s spiral to two concentric circles and then to the circular arrangement is the increased regularity of the distribution of VSs, which is considered undesirable and can be discerned in the sidelobe patterns presented in Figure 9a,b and Figure 10a,b.

The limited impact of transmission sequences on the FWHM metric is expected, since lateral resolution is primarily determined by the transducer’s geometry, focal depth, and steering, parameters that were kept constant throughout all transmission strategies. This explains the absence of statistically significant pairwise differences for FWHM. In contrast, the other metrics are more sensitive to the transmission scheme, as they reflect variations in beam shape and sidelobe behavior. This is particularly evident in the statistical analysis of ISL, which revealed multiple significant pairwise differences between transmission strategies.

As expected, plane wave transmission achieved undesired scores in all metrics, serving its purpose as the lowest baseline. As for DW, when following a circular and two concentric circles arrangement, PSL and ISL showed the best results of all transmissions, including the best clutter rejection. Placing the VSs following a Fermat’s spiral led to the best resolution (FWHM) and contrast even though its PSL and ISL scores were not outstanding, meaning that it has a good handle of the sidelobes, but other distributions were able to reduce them more. Finally, the focused transmission scored a very good CR, with results within 10% of the best, but had high PSL values.

For the proposed annular arrays, our findings indicate that the Fermat’s spiral offers a balanced outcome across metrics, but there is no decisive advantage over the symmetry-preserving VS sets.

Experimental data show that for more complex scanning geometries, such as scenarios involving multiple inclusions in an already echoic medium, the differentiation of structural elements becomes challenging, even with the use of the optimized transmission sequence. Despite this, the targeted wires remained almost equally visible regardless of depth, according to the CR results. We note that the reduced visibility of targets very proximal to the aperture or beyond approximately 8 cm reflects the detection limits of this experimental configuration, not a fundamental limit of the array architecture. At larger depths, spatial resolution and signal-to-noise ratio decrease, but Doppler motion detection may remain feasible. Moreover, even for the wires that could be distinguished, information could be retrieved, but the raw image quality might still impose a challenge for interoperability.

The proposed transducer presents imaging limitations that could be improved in future transducer generations. Its potential in imaging can be enhanced to a certain extent by using DW transmission with 45 VSs following a Fermat’s spiral. Other more conventional arrangements of VSs showed better performance in some aspects, as when going from the circular to the two concentric circles to the Fermat’s spiral arrangement we gain contrast due to an increased resolution at the cost of higher PSL and ISL. As an important finding, for this aperture, the Fermat’s spiral DW outperformed the focused wave transmission when looking at the overall metric performance. Here, the decorrelation of sidelobes from Fermat’s spiral seems more effective than the expected suppression from focused transmission, as a result of the poor steerability that such a transducer geometry offers. Despite its limitations, it establishes a foundational framework for the design and fabrication of cost-effective, compact, 2-D transducers suitable for 3-D ultrasound imaging, which can be seamlessly integrated into wearable devices.

## Figures and Tables

**Table 1:** Typical performance values of the proposed transducer.

Parameter	Value	Unit
Wafer level		
Bias voltage	35	V
Max. voltage (bias + RF)	55	V
Acoustical characterization		
Center frequency	2.7	MHz
Fractional bandwidth *	116	%
Max. pressure ^†^	1.4	MPa
Sensitivity	3.4	MPa/100V RF

* Fractional bandwidth is computed as $FBW_{-3dB}=\frac{f_{h}-f_{l}}{fc}\times 100\%$, where $f_{l}$ and $f_{h}$ are the lower and upper frequencies, respectively, at which the magnitude response drops by 3 dB (half-power) from its peak, and $f_{c}$ is the center frequency. ^†^ Surface pressure when applying a bias voltage of 35 V with an additive radiofrequency pulse amplitude of 20 V.

**Table 2:** MAE for different values of *a*.

Piston Width (*a*) Relative to λ	Mean Absolute Error (MAE)
0.5λ	0.089
1.0λ	0.022
1.5λ	0.012
2.0λ	0.008
2.5λ	0.003
3.0λ	0.002

**Table 3:** Selected $n_{vs}$ for different VS patterns.

Pattern	$n_{\mathrm{vs}}$ at ε-plateau, R=[5,10,15] cm	Selected $n_{\mathrm{vs}}$
Circumference	[40, 36, 40]	40
Two concentric circ.	[24, 36, 36]	36
Fermat’s spiral	[40, 45, 40]	45

**Table 4:** Statistical test *p*-values for different metrics and *R*. RM-ANOVA stands for repeated measurements ANOVA.

Depth	Test	*p*-Value
**FWHM**		
5 cm	RM-ANOVA	<0.001
10 cm	RM-ANOVA	<0.001
15 cm	RM-ANOVA	0.002
**CR**		
5 cm	Friedman	0.009
10 cm	Friedman	0.028
15 cm	Friedman	0.029
**PSL**		
5 cm	RM-ANOVA	<0.001
10 cm	Friedman	0.014
15 cm	RM-ANOVA	0.001
**ISL**		
5 cm	RM-ANOVA	<0.001
10 cm	RM-ANOVA	<0.001
15 cm	RM-ANOVA	0.002

**Table 5:** Results of metric evaluation for each wire.

Depth (z) [mm]	FWHM [mm]	CR [dB]
29.91	1.51	15.57
39.87	6.31	16.92
50.30	6.21	14.19
60.26	2.50	15.39
70.84	6.44	17.26
80.80	-	14.28

## Data Availability

The original contributions presented in this study are included in the article. Further inquiries can be directed to the corresponding author.

## Abbreviations

The following abbreviations are used in this manuscript:

CTG	Cardiotocography
CMUT	Capacitive micromachined ultrasonic transducer
DW	Diverging waves
VS	Virtual source
RSI	Rayleigh–Sommerfeld integral
PSF	Point spread function
CR	Contrast ratio
FWHM	Full width at half-maximum
PSL	Peak sidelobe level
ISL	Integrated sidelobe level
MIP	Maximum Intensity Projection

## Appendix A. Bessel of First Kind Piecewise Approximation

Bessel functions of the first kind $J_{n}\left(x\right)$ are defined as the solutions of the Bessel differential equation [47],

$$x^{2}\frac{d^{2}y}{dx^{2}}+x\frac{dy}{dx}+\left(x^{2}-n^{2}\right)y=0\;.$$

The series expansion for the Bessel function of the first kind of order one, $J_{1}\left(x\right)$, is given by [48]:

$$J_{1}\left(x\right)=\sum_{m=0}^{\infty }\frac{{\left(-1\right)}^{m}}{m!\;(m+1)!}\;{\left(\frac{x}{2}\right)}^{2m+1}\;.$$

If the series is truncated to a finite number of terms, the following polynomial approximation is obtained:

$$J_{1}\left(x\right)=\frac{x}{2}-\frac{1}{1!2!}{\left(\frac{x}{2}\right)}^{3}+\frac{1}{2!3!}{\left(\frac{x}{2}\right)}^{5}-\frac{1}{3!4!}{\left(\frac{x}{2}\right)}^{7}+\ldots$$

The series converges for all *x* and produces progressively better accuracy with more terms included. The implemented polynomial approximation ensures accuracy while keeping the computational effort low. For this approximation, multiple cases are considered:

1. Very small |x|$\left(|x|\leq 10^{-7}\right)$.
  Here $J_{1}\left(x\right)\approx x/2$ already gives machine precision accuracy, avoiding divisions by very small numbers in the polynomial.
2. Mid range |x|$\left(10^{-7}<|x|\leq 4\right)$.
  A polynomial expansion in odd powers of *x* is used,
  $$J_{1}\left(x\right)\;\approx \;c_{1}x+c_{3}x^{3}+c_{5}x^{5}+c_{7}x^{7}+c_{9}x^{9},$$
  where the coefficients $c_{1},c_{3},\ldots$ are chosen to match the Maclaurin series for $J_{1}\left(x\right)$ up to |x|≈4.
3. Large |x|(|x|>4).
  The standard asymptotic form of the Bessel function is used:
  $$J_{1}\left(x\right)\;\approx \;\sqrt{\frac{2}{\pi |x|}}\;\cos\;\left(\left|x\right|-\frac{3\pi }{4}\right),\;\left|x\right|\to \infty .$$

Finally, a sign correction is applied if x<0,

$$J_{1}\left(-x\right)=-J_{1}\left(x\right),$$

since $J_{1}$ is an odd function.

## Appendix B. Computation of $\alpha_{v_{\max}}$

The directivity, *D*, is given by

$$D\left(\alpha ,\varphi \right)\approx \mathrm{sinc}\left(\frac{kW}{2}\sin\alpha \cos\varphi \right)\mathrm{sinc}\left(\frac{kL}{2}\sin\alpha \sin\varphi \right),$$

where α and φ are the elevation and azimuthal angle, respectively, within the plane of the element measured from the tangential axis; *W* is the width of the element, *L* is the length of the element, and *k* is the wave number [49]. The first null point of either of the sinc functions indicates the maximum angle for element transmission, $\alpha_{max}$,

$$\frac{kW}{2}\sin\alpha_{max}\cos\varphi =\pi ,\;\frac{kL}{2}\sin\alpha_{max}\sin\varphi =\pi$$

Substituting k=2π/λ, an $\alpha_{max}$ that holds for all φ is given by

$$\alpha_{max}=\arcsin\left(\frac{\lambda }{\max(W,L)}\right).$$

Plugging in the values corresponding to this study,

$$\alpha_{max}\approx 32.90{}^{\circ},$$

so

$$\alpha_{v_{max}}=2\alpha_{max}\approx 65.80{}^{\circ}.$$

## References

[1] Soma-Pillay P., Nelson-Piercy C., Tolppanen H., Mebazaa A. (2016). Physiological changes in pregnancy. Cardiovasc. J. Afr..

[2] Qiu J., Chen L., Wang X., Zhu W. (2022). Early-pregnancy maternal heart rate is related to gestational diabetes mellitus (GDM). Eur. J. Obstet. Gynecol. Reprod. Biol..

[3] Force U.S.P.S.T., Bibbins-Domingo K., Grossman D.C., Curry S.J., Barry M.J., Davidson K.W., Doubeni C.A., Epling J.W., Kemper A.R., Krist A.H. (2017). Screening for Preeclampsia: US Preventive Services Task Force Recommendation Statement. JAMA.

[4] Lyons E.R., Bylsma-Howell M., Shamsi S., Towell M.E. (1979). A scoring system for nonstressed antepartum fetal heart rate monitoring. Am. J. Obstet. Gynecol..

[5] Fanelli A., Ferrario M., Piccini L., Andreoni G., Matrone G., Magenes G., Signorini M.G. Prototype of a wearable system for remote fetal monitoring during pregnancy. Proceedings of the 2010 Annual International Conference of the IEEE Engineering in Medicine and Biology.

[6] (2021). American College of Obstetricians and Gynecologists, Committee on Obstetric Practice, Society for Maternal-Fetal Medicine. Indications for outpatient antenatal fetal surveillance: ACOG Committee Opinion, Number 828. Obstet. Gynecol..

[7] Kauffmann T., Silberman M. (2023). Fetal Monitoring.

[8] Ahmed M.R., Newby S., Potluri P., Mirihanage W., Fernando A. (2024). Emerging Paradigms in Fetal Heart Rate Monitoring: Evaluating the Efficacy and Application of Innovative Textile-Based Wearables. Sensors.

[9] Ayres-de Campos D. (2018). Electronic fetal monitoring or cardiotocography, 50 years later: What’s in a name?. Am. J. Obstet. Gynecol..

[10] Walton J.R., Peaceman A.M. (2012). Identification, Assessment and Management of Fetal Compromise. Clin. Perinatol..

[11] Hamelmann P., Vullings R., Kolen A.F., Bergmans J.W.M., van Laar J.O.E.H., Tortoli P., Mischi M. (2020). Doppler Ultrasound Technology for Fetal Heart Rate Monitoring: A Review. IEEE Trans. Ultrason. Ferroelect. Freq. Control..

[12] Adam J. (2012). The Future of Fetal Monitoring. Rev. Obstet. Gynecol..

[13] Huang H., Wu R.S., Lin M., Xu S. (2024). Emerging Wearable Ultrasound Technology. IEEE Trans. Ultrason. Ferroelectr. Freq. Control.

[14] Hu H., Huang H., Li M., Gao X., Yin L., Qi R., Wu R.S., Chen X., Ma Y., Shi K. (2023). A wearable cardiac ultrasound imager. Nature.

[15] Song P., Andre M., Chitnis P., Xu S., Croy T., Wear K., Sikdar S. (2023). Clinical, Safety, and Engineering Perspectives on Wearable Ultrasound Technology: A Review. IEEE Trans. Ultrason. Ferroelectr. Freq. Control.

[16] Wang C., Chen X., Wang L., Makihata M., Liu H.-C., Zhou T., Zhao X. (2022). Bioadhesive ultrasound for long-term continuous imaging of diverse organs. Science.

[17] Leung K.-Y. (2021). Applications of Advanced Ultrasound Technology in Obstetrics. Diagnostics.

[18] Steiner H., Staudach A., Spitzer D., Schaffer H. (1994). Three-dimensional ultrasound in obstetrics and gynaecology: Technique, possibilities and limitations. Hum. Reprod..

[19] Roux E., Varray F., Petrusca L., Cachard C., Tortoli P., Liebgott H. (2018). Experimental 3-D ultrasound imaging with 2-D sparse arrays using focused and diverging waves. Sci. Rep..

[20] De Hoop H., Vermeulen M., Schwab H.M., Lopata R.G.P. (2023). Coherent Bistatic 3-D Ultrasound Imaging Using Two Sparse Matrix Arrays. IEEE Trans. Ultrason. Ferroelectr. Freq. Control.

[21] Masoumi M.H., Kaddoura T., Zemp R.J. (2023). Costas Sparse 2-D Arrays for High-Resolution Ultrasound Imaging. IEEE Trans. Ultrason. Ferroelectr. Freq. Control.

[22] Jensen J.A., Schou M., Jørgensen L.T., Tomov B.G., Stuart M.B., Traberg M.S., Taghavi I., Øygaard S.H., Ommen M.L., Steenberg K. (2022). Anatomic and Functional Imaging Using Row–Column Arrays. IEEE Trans. Ultrason. Ferroelectr. Freq. Control.

[23] Awad S.I., Yen J.T. (2009). 3-D Spatial Compounding Using a Row-Column Array. Ultrason. Imaging.

[24] Zhang J., Huang C., Lok U.W., Dong Z., Liu H., Gong P., Song P., Chen S. (2025). Enhancing Row-Column Array (RCA)-Based 3D Ultrasound Vascular Imaging With Spatial-Temporal Similarity Weighting. IEEE Trans. Med. Imaging.

[25] Morton C., Lockwood G. Theoretical assessment of a crossed electrode 2-D array for 3-D imaging. Proceedings of the IEEE Symposium on Ultrasonics.

[26] Ramalli A., Boni E., Savoia A.S., Tortoli P. (2015). Density-tapered spiral arrays for ultrasound 3-D imaging. IEEE Trans. Ultrason. Ferroelectr. Freq. Control.

[27] Ramalli A., Boni E., Giangrossi C., Mattesini P., Dallai A., Liebgott H., Tortoli P. (2021). Real-Time 3-D Spectral Doppler Analysis With a Sparse Spiral Array. IEEE Trans. Ultrason. Ferroelectr. Freq. Control.

[28] Vos H.J., Boni E., Ramalli A., Piccardi F., Traversi A., Galeotti D., Noothout E.C., Daeichin V., Verweij M.D., Tortoli P. Sparse Volumetric PZT Array with Density Tapering. Proceedings of the 2018 IEEE International Ultrasonics Symposium (IUS).

[29] Martínez-Graullera O., Martín C.J., Godoy G., Ullate L.G. (2010). 2D array design based on Fermat spiral for ultrasound imaging. Ultrasonics.

[30] Turnbull D.H., Foster F.S. Two-dimensional transducer arrays for medical ultrasound: Beamforming and imaging (Invited Paper). Proceedings of the New Developments in Ultrasonic Transducers and Transducer Systems.

[31] Diarra B., Robini M., Tortoli P., Cachard C., Liebgott H. (2013). Design of Optimal 2-D Nongrid Sparse Arrays for Medical Ultrasound. IEEE Trans. Biomed. Eng..

[32] Roux E., Ramalli A., Tortoli P., Cachard C., Robini M.C., Liebgott H. (2016). 2-D Ultrasound Sparse Arrays Multidepth Radiation Optimization Using Simulated Annealing and Spiral-Array Inspired Energy Functions. IEEE Trans. Ultrason. Ferroelectr. Freq. Control.

[33] Ortiz S.H.C., Chiu T., Fox M.D. (2012). Ultrasound image enhancement: A review. Biomed. Signal Process. Control..

[34] Lokesh B., Thittai A.K. (2019). Diverging beam transmit through limited aperture: A method to reduce ultrasound system complexity and yet obtain better image quality at higher frame rates. Ultrasonics.

[35] Montaldo G., Tanter M., Bercoff J., Benech N., Fink M. (2009). Coherent plane-wave compounding for very high frame rate ultrasonography and transient elastography. IEEE Trans. Ultrason. Ferroelectr. Freq. Control.

[36] Hasegawa H., Kanai H. (2011). High-frame-rate echocardiography using diverging transmit beams and parallel receive beamforming. J. Med. Ultrason..

[37] de Hoop H., Petterson N.J., van de Vosse F.N., van Sambeek M.R.H.M., Schwab H.M., Lopata R.G.P. (2020). Multiperspective Ultrasound Strain Imaging of the Abdominal Aorta. IEEE Trans. Med. Imaging.

[38] Papadacci C., Pernot M., Couade M., Fink M., Tanter M. (2014). High-contrast ultrafast imaging of the heart. IEEE Trans. Ultrason. Ferroelectr. Freq. Control.

[39] van Schaijk R., in ’t Zandt M., Robaeys P., Slotboom M., Klootwijk J., Bekkers P. Reliability of collapse mode CMUT. Proceedings of the 2023 IEEE International Ultrasonics Symposium (IUS).

[40] Herickhoff C.D., van Schaijk R. (2023). cMUT technology developments. Z. Fur Med. Phys..

[41] Jensen J.A. (1997). Field: A Program for Simulating Ultrasound Systems: 10th Nordic-Baltic Conference on Biomedical Imaging. Med. Biol. Eng. Comput..

[42] Jensen J.A. (2001). Users’ Guide for the Field II Program. 3.30 ed.

[43] McGough R.J. (2004). Rapid calculations of time-harmonic nearfield pressures produced by rectangular pistons. J. Acoust. Soc. Am..

[44] Cigier A., Varray F., Garcia D. (2022). SIMUS: An open-source simulator for medical ultrasound imaging. Part II: Comparison with four simulators. Comput. Methods Programs Biomed..

[45] Treeby B.E., Cox B.T. (2010). k-Wave: MATLAB toolbox for the simulation and reconstruction of photoacoustic wave fields. J. Biomed. Opt..

[46] Encino K., Panduro M.A., Reyna A., Covarrubias D.H. (2022). Novel Design Techniques for the Fermat Spiral in Antenna Arrays, for Maximum SLL Reduction. Micromachines.

[47] Abramowitz M., Stegun I.A. (1965). Handbook of Mathematical Functions: With Formulas, Graphs, and Mathematical Tables.

[48] Harrison J. Fast and Accurate Bessel Function Computation. Proceedings of the 2009 19th IEEE Symposium on Computer Arithmetic.

[49] Szabo T.L. Array Beamforming. Diagnostic Ultrasound Imaging: Inside Out.

